# Evaluation of fish skin as a biological dressing for metacarpal wounds in donkeys

**DOI:** 10.1186/s12917-020-02693-w

**Published:** 2020-12-03

**Authors:** Ahmed Ibrahim, Mahmoud Soliman, Saber Kotb, Magda M. Ali

**Affiliations:** 1 Veterinary Teaching Hospital, Faculty of Veterinary Medicine, Assiut University, Assiut, 71526 Egypt; 2 Department of Veterinary Pathology and Clinical Pathology, Faculty of Veterinary Medicine, Assiut University, Assiut, 71526 Egypt; 3 Department of Animal and Poultry Hygiene, and Environmental Sanitation, Faculty of Veterinary Medicine, Assiut University, Assiut, 71526 Egypt; 4 Department of Surgery, Anesthesiology, and Radiology, Faculty of Veterinary Medicine, Assiut University, Assiut, 71526 Egypt

**Keywords:** Fish skin, Tilapia, Silver nanoparticles, Dressing, Wounds, Donkeys

## Abstract

**Background:**

The use of biological dressings has recently emerged in the management of burns and wounds. The aim of the present study was to evaluate the Nile tilapia skin as a biological dressing for full-thickness cutaneous metacarpal wounds in donkeys. The study was conducted on nine clinically healthy donkeys (*n* = 9). Here, fish skin dressings were obtained from fresh Nile tilapia *(Oreochromis niloticus* and sterilized by immersion in silver nanoparticles (AgNPs) solution for 5 min, with no change in collagen content. Bilateral, circular full-thickness excisional skin wounds (2 cm in diameter) were created on the dorsal aspect of the mid-metacarpals of each donkey. Wounds on the right metacarpals (treated wounds, *n* = 9) were dressed with sterile fish skins, while wounds on the left metacarpals (control wounds, *n =* 9) were dressed with sterile non-adherent dressing pads without any topical applications. Wound dressings were changed weekly. Wounds were evaluated microbiologically, grossly, and histologically on days 7, 14, and 21 post-wound inductions.

**Results:**

Fish skin-dressed wounds showed a significant (*P < 0.0001*) reduction in microbial counts (Total viable bacterial count, Staphylococcal count, and Coliform count), a significant (*P < 0.0001*) decrease in the wound size, and a significant reduction (*P < 0.0001*) in the epithelial gap compared to the untreated wounds. No frequent dressing changes were needed.

**Conclusions:**

Fish skin dressing accelerated the wound healing process and efficiently inhibited the local microbial activity and exuberant granulation tissue formation suggesting its reliable and promising application for metacarpal wounds of donkeys.

## Background

Wounds of the distal limbs of equines are common and represent more than 60% of all wounds. These wounds are more problematic than others located elsewhere in the body due to its high susceptibility to contamination, the poor healing nature, and high tendency to form exuberant granulation tissue [1].

Biological wound dressings are that derived from natural resources and are known for their biocompatibility and biodegradability [2]. Several biological materials have been used for wound dressing in equines including: the porous bovine collagen membrane, keratinocyte collagen membrane, split-thickness allogeneic skin, allogeneic peritoneum, and xenogeneic porcine small intestinal submucosa [3–5].

Fish skin contains collagen type I and III in large quantities [6], a protein that is potential to promote wound healing [7, 8]. Fish skin has been used for the first time as a biological wound dressing for the second- and third-degree burns of humans in Brazil. No dressing changes were required as frequently as the gauze. Moreover, fish skin dressing enhanced the wound healing process and reduced the need for pain medications [9].

Fish skin has been used in elsewhere cases as an occlusive wound dressing for burn management in humans [10, 11] and animals (pony, mountain lion, and bears) [12, 13] demonstrating a complete re-epithelialization in a brief time faster than expected. However, the treatment with the fish skin is still in the experimental stage [11]. Moreover, available literature lacks detailed studies regarding the use of fish skin as an equine wound dressing. Hence, the present study aimed to evaluate the Nile tilapia *(Oreochromis niloticus)* skin as a biological dressing for the full-thickness cutaneous metacarpal wounds in donkeys (*Equus asinus*).

## Results

### Microbiological evaluation of AgNPs-treated fish skin

All bacteriological swabs of AgNPs treated fish skin strips showed no microbial growth on the four bacteriological media (the Plate Count Agar, Mannitol Salt Agar, MacConkey Agar, and Dextrose Agar).

### Histological and histochemical evaluation of AgNPs-treated fish skin

**Fig. 1:**
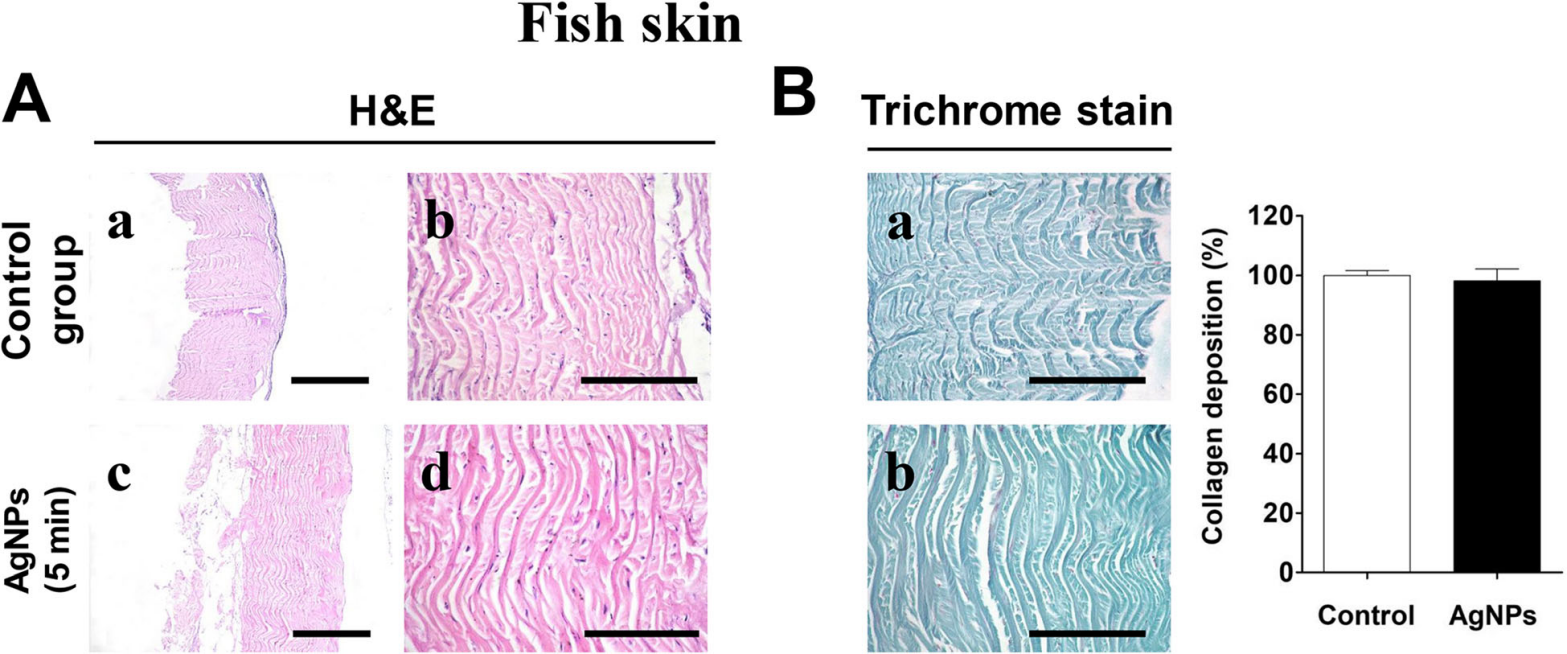
Histological and histochemical evaluation of fish skin sterilized with silver nanoparticles (AgNPs). **a** Hematoxylin and eosin stained sections. **b** Gomori’s trichrome stained sections for collagen. The scale bars in panel A (a and c = 100 μm, b and d = 50 μm) and in panel B = 50 μm

Histological analysis of the fish skin treated or not with the AgNPs revealed that the collagen fibers were compactly arranged, well organized, and distributed in a parallel pattern without disaggregation (Fig. 1a). Staining of the collagen fibers with Gomori’s trichrome stain showed the well organization of the collagen fibers and the preservation of the collagen intensity in both conditions (Fig. 1b).

### Microbiological evaluation of wounds

Data presented in Table 1 showed the microbial contamination (Total viable bacterial count, Staphylococcal count, and Coliform count) of the wounds dressed by AgNPs-treated fish skin (treated wounds) versus control ones. The statistical analysis revealed that there was a significant difference (*P < 0.0001*) between the fish skin dressed wounds and control wounds at different contact times (7, 14, and 21 days post-wound induction). The highest reduction percentage produced the highest antibacterial activity against Total viable bacterial count, Staphylococcal count, and coliform count of the treated wounds ranged from 83.83 to 100% from the 7th day to the 21st day post-wound induction. On the contrary, the mean value of microbial contaminants of untreated wounds increased by increasing the wound age.

**Table 1:** Microbiological evaluation of fish skin treated wounds

Treatment	Time (day)	Microbial counts (cm^**2**^) (Mean ± SE)							
		Aerobic bacterial count		Staphylococcal count		Coliform count		Yeast and mold count	
			Reduction %		Reduction %		Reduction %		Reduction %
**Control**	0	2.63^a^ × 10^2^ ± 2.025		1.5^a^ × 10^2^ ± 1.80		1.5 ^a^ × 10^2^ ± 1.23		–	–
	7	355.25^c^ ± 1.31	−35.07%	180.00^d^ ± 1.04	−20.0%	171.2^d^ ± 1.32	−14.13%	–	–
	14	360.3^b^ ± 13.33	−38.46%	196.0^d^ ± 1.23	−30.66%	238.75^d^ ± 1.97	−59.16%	–	–
	21	371.66^b^ ± 1.143	−41.31%	198.5^d^ ± 1.122	−32.33%	283.33^d^ ± 1.20	−88.8%	–	–
**Fish skin dressing**	0	2.6^a^ × 10^2^ ± 2.013		2^a^ × 10^2^ ± 1.69		1.3^a^ × 10^2^ ± 1.03		–	–
	7	23.33^b^ ± 0.66	93.15%	18.00^b^ ± 0.64	91.00%	22.66^b^ ± 0.64	83.83%	–	–
	14	0.25^c^ ± 0.25	99.93%	6.25^c^ ± 0.625	96.87%	00.00^c^ ± 00.00	100%	–	–
	21	00.00^c^ ± 00.00	100%	00.00^c^ ± 00.00	100%	00.00^c^ ± 00.00	100%	–	–

a, b, c, and d values with no common superscript differ significantly (*P < 0.05*)

### Gross evaluation of wounds

**Fig. 2:**
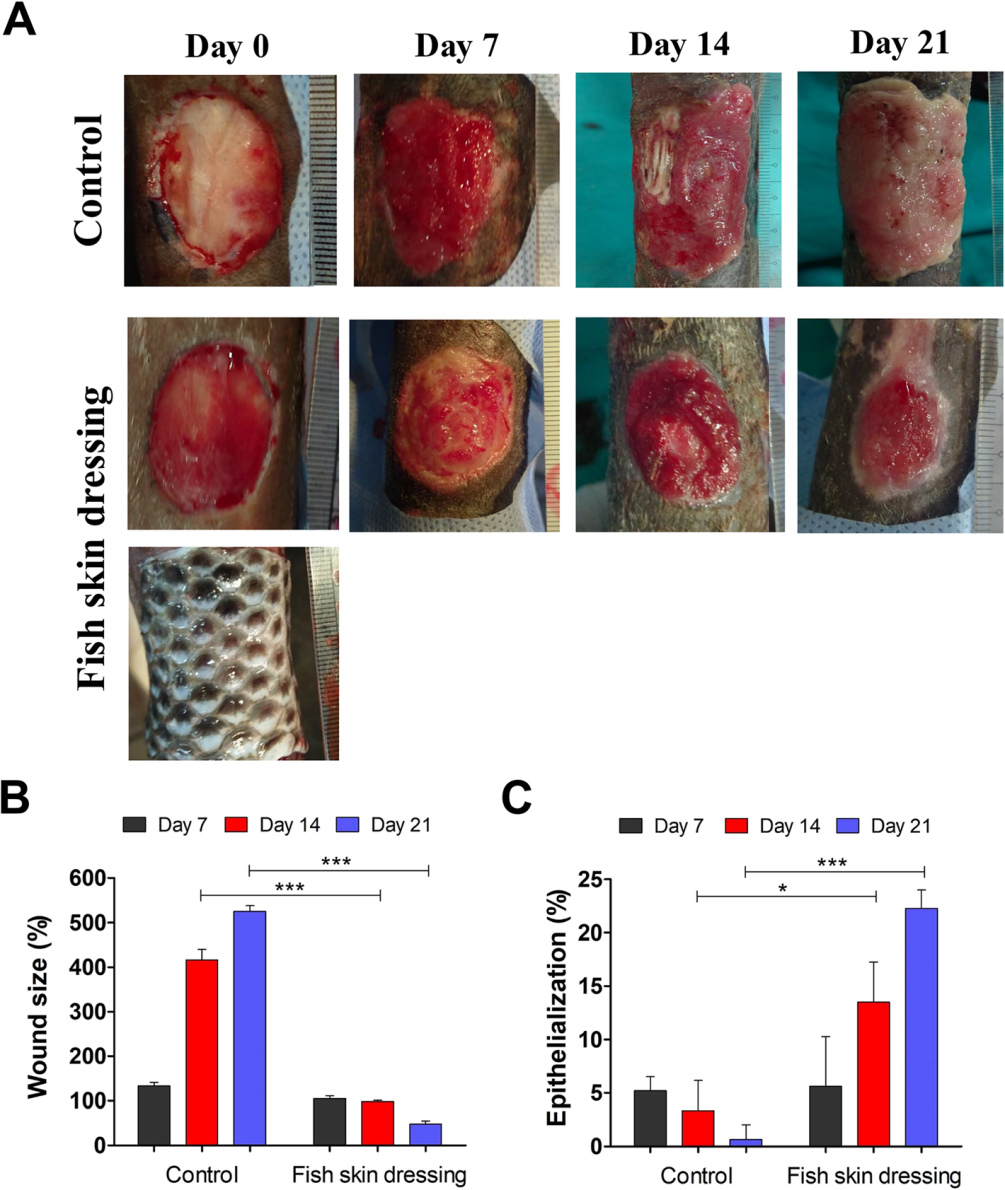
Gross examination of the metacarpal wounds in donkeys dressed with or without fish skin. **a** Representative photographs of the skin wounds of the control- or fish skin-dressed groups on days 0, 7, 14, and 21post-wound inductions. Ruler units are mm. The wound size (**b**) and the epithelialization (**c**) were measured using the ImageJ software. *p < 0.05, **p < 0.001, ***p < 0.0001

The fish skin dressing improved the metacarpal wound healing grossly. The metacarpal wounds dressed with fish skin initially increased in size on day 7 post-wound inductions (Fig. 2a and b). However, the percentage of wound size was significantly (*P < 0.0001*) decreased over the experimental time (Fig. 2a and b), and appeared more pronounced on day 21 post-wound induction (Fig. 2a and b). The wounds of the control group gradually increased in size between days 7 up to 21 post-wound inductions, reached 4–5 times that of the original wound size, and appeared elevated more than the surrounding skin due to the hypergranulation tissue formed in the wound site (Fig. 2a and b). The rate of epithelialization was gradually increased in the fish skin dressed wounds compared to the control wounds (Fig. 2c).

### Histological evaluation of wounds

**Fig. 3:**
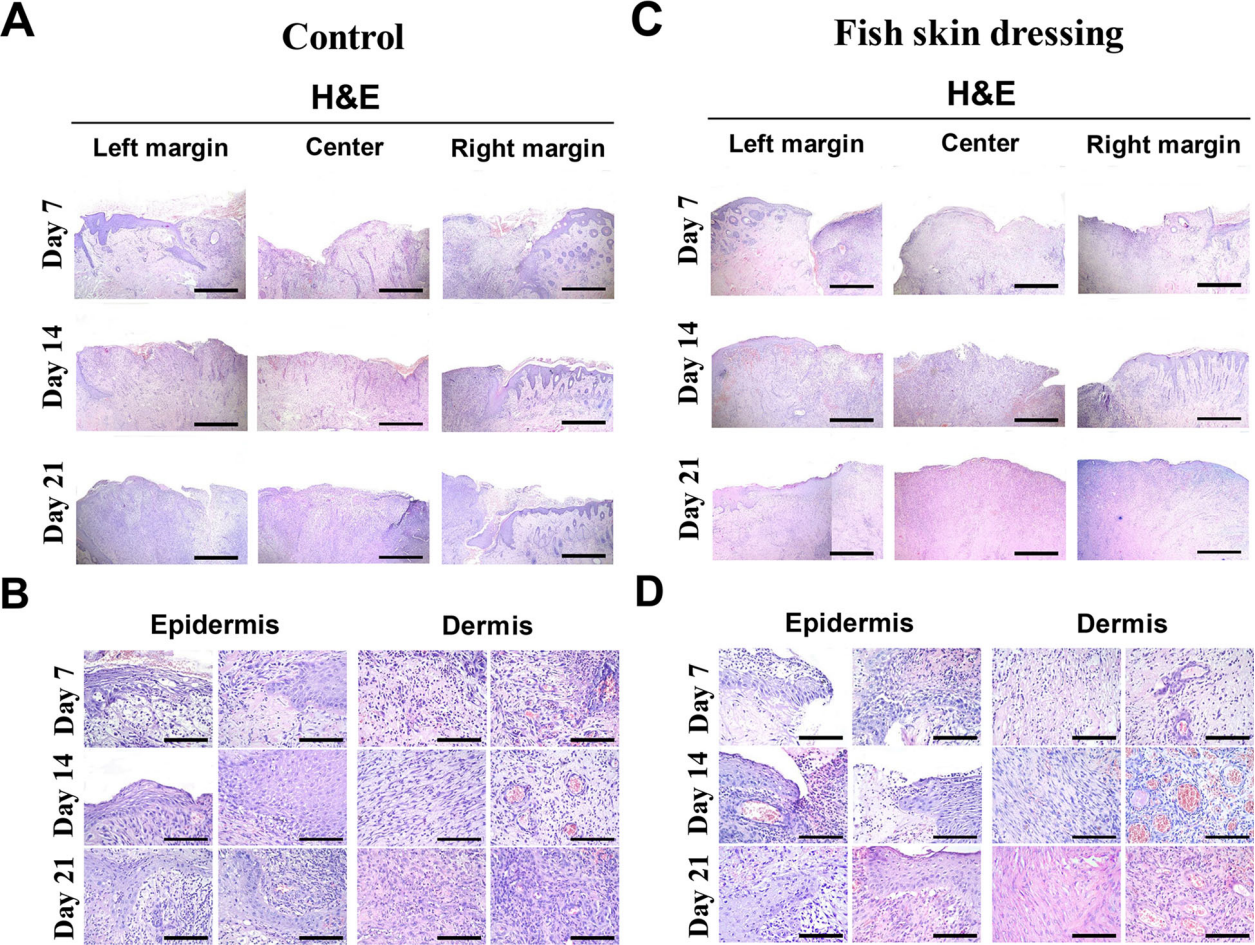
Histological evaluation of the metacarpal wounds in donkeys dressed with or without fish skin. Hematoxylin and eosin stained sections from the control- (**a**) and (**b**) or fish skin-dressed (**c**) and (**d**) wounds harvested on days 7, 14, and 21 post-wound inductions. Scale bars in A and C = 100 μm and in B and D = 50 μm

**Fig. 4:**
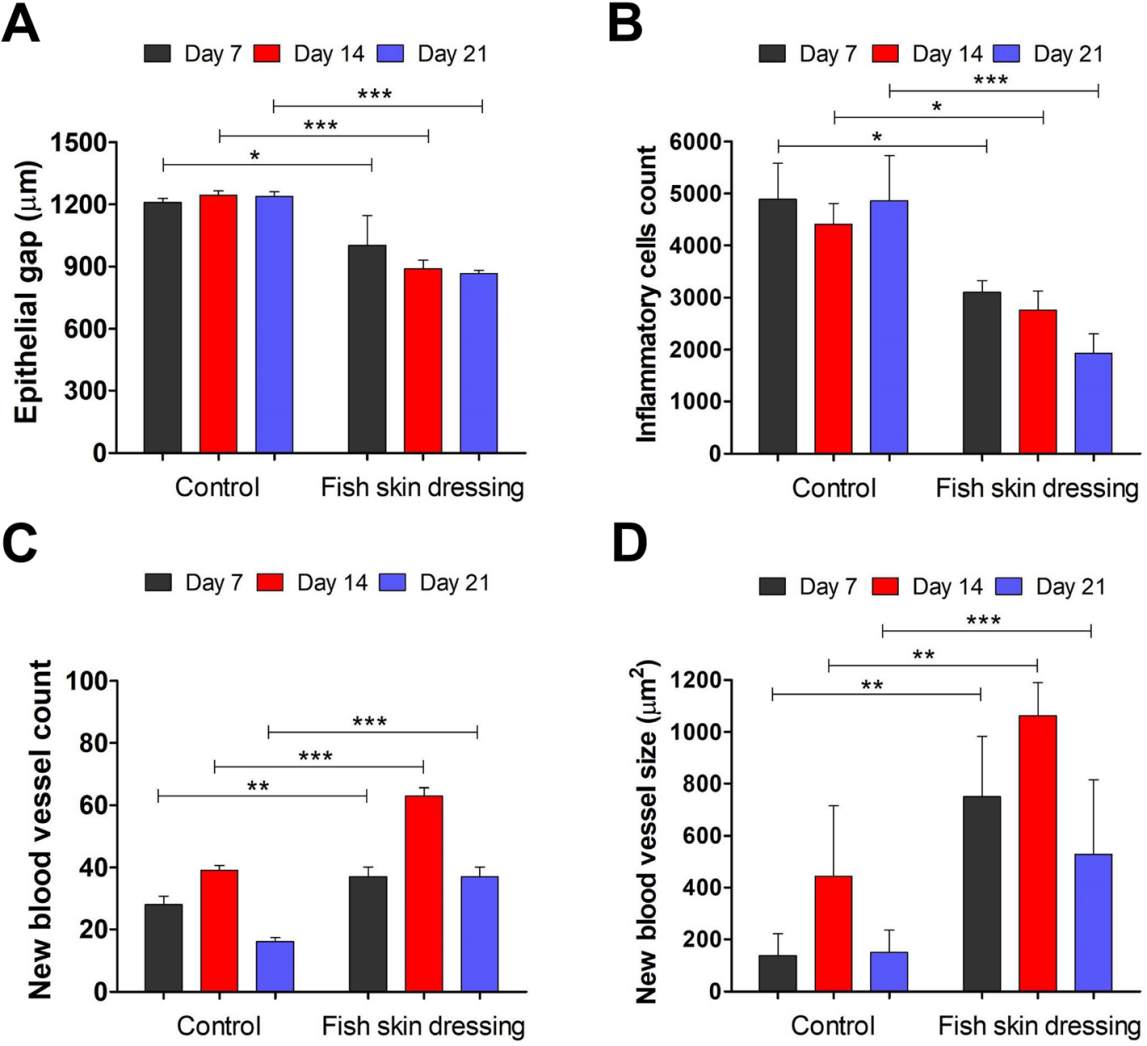
Evaluation of healing parameters in the metacarpal wounds in donkeys dressed with or without fish skin. **a** The epithelial gap between the two margins of the wound and (**b**) the inflammatory cells count in the wound site were measured using Imag J software. **c** The number of new blood vessels was counted in 5 images/wound sample. **d** The size of new blood vessels was measured using the ImageJ software. *p < 0.05, **p < 0.001, ***p < 0.0001

In the epidermis, the epidermal hyperplasia at the wound edges was more pronounced in the wounds dressed with the fish skin compared with the control ones (Fig. 3), evidenced by the significant (*P < 0.0001*) reduction in the epithelial gap in the fish skin dressed wounds (Fig. 4a). In contrast, the epithelial gap was slightly increased in a time-dependent manner in control wounds (Fig. 4a).

In the dermis, a large number of inflammatory cells (neutrophils and macrophages) appeared in the wound site in the control group, which remained at a high level throughout the experiment (Figs. 3c and 4b). However, the fish skin dressed wounds were largely infiltrated with the inflammatory cells on day 7 post-wound induction, which greatly decreased with time, on days 14 and 21 post-wound induction (Figs. 3d and 4b). The formation of new blood vessels was highly increased in number in the fish skin dressed wounds compared with the control wounds, peaking on day 14 and subsequently decreasing on day 21 post-wound induction (Figs. 3b and c and 4c). This was accompanied with a similar pattern of increase in the size of the blood vessels in the wounds dressed with the fish skin as compared with the control wounds (Figs. 3b and c and 4d).

### Histological evaluation of the granulation tissue

**Fig. 5:**
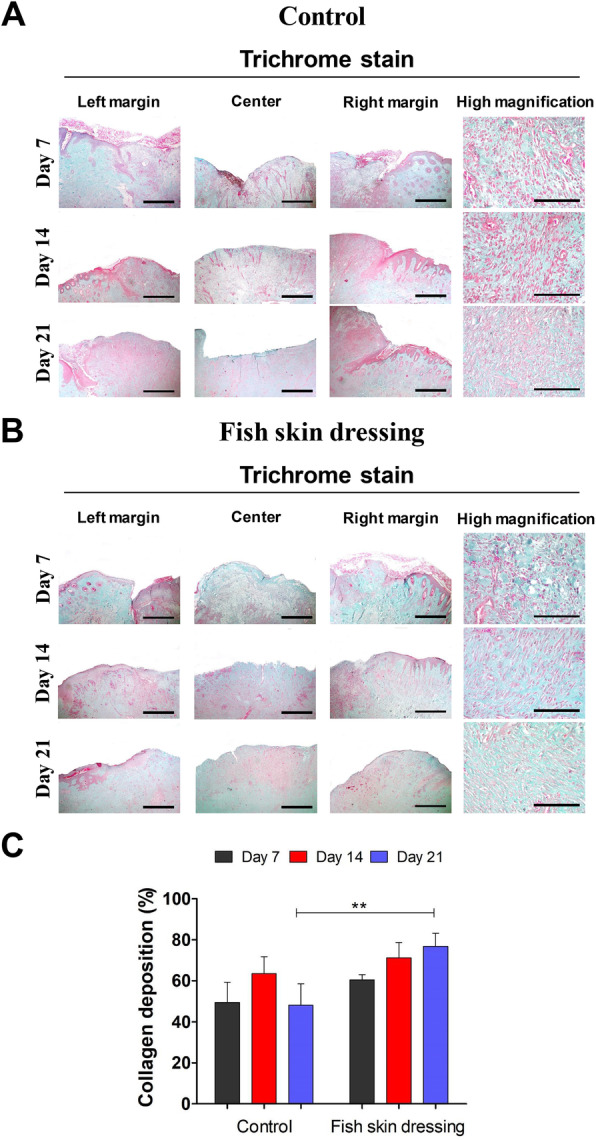
Histochemical evaluation of the metacarpal wounds in donkeys dressed with or without fish skin. **a** and **b** Gomori’s Trichrome stained sections from the control- (**a**) or fish skin-dressed (**b**) wounds harvested on days 7, 14, and 21 post-wound inductions. Scale bars = 100 μm and in high magnification panels = 50 μm. **c** The collagen deposition was quantified using the ImageJ software. The results are expressed as a percentage from the total number of pixels and are normalized to the control group. *p < 0.05, **p < 0.001, ***p < 0.0001

The stained wound specimens with Gomori’s trichrome stain demonstrated proliferation of the fibroblast in the wound area with the formation of granulation tissue (Fig. 5a and b). On days 7 and 14 post-wound induction, there was no significant (*P > 0.05*) difference in the collagen intensity between the control and fish skin dressed groups (Fig. 5c). However, on day 21 post-wound induction, the wounds dressed with the fish skin showed intensive staining of the fibers and a significant increase in the collagen deposition (Fig. 5b and c).

## Discussion

The clinical findings of the earlier clinical trials following the use of the fish skin as a biological dressing for burn management in human [10, 11] and animals [12, 13] was the motivation to carry out the current study. Therefore, the present study evaluated the effect of fish skin on the wound healing process. The present study demonstrated that fish skin dressing of the metacarpal wounds of donkeys promoted wound epithelialization and accelerated the wound healing time. Moreover, it was efficient in inhibition of wound contaminants, as well as, exuberant granulation tissue formation, and did not require frequent dressing changes.

Nile tilapia fish skin has high collagen content more than in other skins [6, 11]. Collagen is the biomaterial that is potential for wound repair [14–16]. This role has been explained on the basis of its hemostatic effect and the scaffold role for fibroblastic proliferation [14]. This was a strong force drive to use the tilapia skin as a xenograft biological dressing for human burn management [10, 11]. Other biological dressings such as bovine peritoneal and amniotic membrane and porcine skin and pericardium and frog skin may have some concerns such as the risk of disease transmission, low availability, or religious beliefs [11, 17].

Tilapia skin has normal non-infectious microbiota [11]. However, it may receive different microorganisms from the water environment [18, 19]. Therefore, the first challenge in the current study was to develop a method for sterilization of the tilapia skin that has to be efficient on microbial reduction without altering its collagen content. This was successfully achieved by immersion of the tilapia skin strips in the silver nanoparticles solution for five minutes with rapid and complete reduction of the microbial growth and preservation of the collagen fibers of the fish skin. Silver nanoparticles are progressively being used in treating and preventing bacterial colonization infections [20, 21]. Chlorhexidine 2%, as well as, radiation at 25 and 30 kGy were efficient methods for sterilization of tilapia skin [6]. However, these methods of sterilization seemed to be relatively time-consuming with adverse effect on the arrangement and content of the collagen fibers of the fish skin.

Metacarpal wounds of donkeys were chosen as a model in the present study as they are the most challengeable ones. Its high tendency to develop hypergranulation tissue, poor vascularization, and high risk of contamination due to its proximity to the floor contribute to lower healing rates than in wounds elsewhere [1]. It was taken into consideration to create the wounds under the effect the intravenous anesthesia rather than local infiltration anesthesia because of the probable adverse effect of the local anesthetic on the wound healing [22]. Also, no antibiotic or anti-inflammatory was used as they could fog wound healing evaluation [23]. Afifah et al. [24] have reported that the wound healing process does not cause pain. Tilapia skin dressing has been exerted anti-nociceptive properties in other studies [10, 11, 17]. This may be attributed to the occlusive and adherent nature of the fish skin as a biological dressing [4, 10].

Fish skin dressings were changed every week. This was in accordance with Lima-Jinior et al. [11] who have been found that fish skin dressings did not need to be changed frequently as the gauze dressing, as well as, it can stay as long as 10 days. Reducing the wound dressing times may alleviate pain and stress associated with dressing change each time especially in chronic wounds.

There was a significant (*P < 0.0001*) reduction in the microbial contamination of the tilapia skin dressed wounds achieving complete inhibition of microorganism growth on 21 days post-wound induction. This may be attributed to the fact that fish skin has antimicrobial properties, therefore, can combat local infection [25–28]. Consistently, the wound area dressed with fish skin was infiltrated with inflammatory cells (neutrophils and macrophages) as early as day 7 post-wounding. These inflammatory cells are important to clean up the wound site from cell debris and infectious agents to enhance the healing process [29, 30]. The influx of inflammatory cells was gradually decreased at days 14 and 21 as the fibroblasts became more abundant.

On the other hand, these inflammatory cells produce a number of chemotactic and growth factors that enhance the epithelialization, formation of new blood vessels, fibroblast proliferation, and collagen formation [29–33]. The re-epithelialization of the wound is largely depending on the presence of the basement membrane, which acts as a scaffold for the migration of basal cells from the wound edges, and on numbers of growth factors released by epithelial cells, endothelial cells, and fibroblasts [29, 34, 35]. Moreover, the application of the tilapia skin dressing kept the wounds moist and hydrated as an occlusive dressing maintaining the correct tissue humidity, which is essential for optimizing and faster epithelialization [10]. Our data showed that fish skin dressing allowed the ingrowth of the epithelial cells, resulting in a decrease in wound size and the epithelial gap between the wound margins.

The formation of new blood vessels in the wound area is crucial for the healing process, to bring nutrients, immune cells, and oxygen to the wound and remove the waste products [36, 37]. Therefore, the fish skin dressed wounds had an increase in the number and size of newly formed blood vessels, indicating that the wound healing might be partially regulated through the blood vessels formation.

Fish skin dressing is also rich in collagen and a number of amino acids, such as proline and alanine, which enhance the proliferation of fibroblasts, granulation tissue formation, and collagen synthesis in the wound [6, 11, 38–40]. The fish skin dressed wounds had minimal collagen during the early stage of the wound healing because the wounds were recently inflamed and at the beginning of the proliferation. However, the intensity of collagen increased at 21 days post-wounding because of the maturation of the granulation tissue with densely packed collagen.

## Conclusions

Fish skin dressing was a reliable treatment for metacarpal wounds of donkeys. The fish skin dressing accelerated the wound healing process and efficiently inhibited the local microbial activity, as well as, exuberant granulation tissue formation. No frequent dressing changes were needed. However, further studies are still needed on a large scale sample of animals, as well as, infected, diabetic, and large-sized wounds to answer remained questions and unclear mechanisms.

## Methods

### Ethical approval

The National Ethical Committee of The Faculty of Veterinary Medicine, Assiut University, Assiut, Egypt, has approved all the procedures in this study in accordance with the Egyptian bylaws and OIE animal welfare standards for animal care and use in research and education.

### Animals

The present study was conducted on nine clinically healthy adult donkeys (*Equus asinus*) (*n* = 9) (4 males and 5 non-pregnant, non-lactating females) with normal blood cell count (red blood cells (T/L): 5.7 ± 0.2, hemoglobin (gm/L): 105 ± 3, packed cell volume (%): 35 ± 1, white blood cells (G/L): 11 ± 0.7, and platelets count (× 10^3^/μL): 273 ± 8) [41], aged between 2 and 3 years and weighing 100–110 kg. Donkeys were obtained from the Experimental Animal House, Veterinary Teaching Hospital, Faculty of Veterinary Medicine, Assiut University, Assiut, Egypt. Donkeys were housed in a standard stable with ad libitum access to feed and water. Donkeys were allocated randomly into three groups (3 donkeys for each, *n* = 3): group 7 (G7), group 14 (G14), and group 21 (G21) based on the evaluation time of wounds on days 7, 14, and 21 post-wound induction, respectively.

### Obtaining of fish skin

The fish skin was collected from fresh Nile tilapia *(Oreochromis niloticus)* (weigh: 720 ± 40 g; standard length: 23 ± 3 cm), obtained from The Aquatic Medicine Unit’s tanks, Faculty of Veterinary Medicine, Assiut University, Assiut, Egypt*.* The fish were euthanized physically by decapitation according to The American Association of Zoo Veterinarians (AAZV) Guidelines for euthanasia [42]. Fresh skin samples were dissected from the underlying tissues after removal of fish scales and then were divided into strips (5 × 5 cm) in sterile normal saline. Fish skin strips were subjected to a process of sterilization, as described below. The sterilized fish skin was subjected to microbiological and histopathological assessment before approving it for wound dressing.

### Sterilization of fish skin

Fish skin strips were sterilized by immersion in silver nanoparticles (AgNPs) solution (25 μg/mL) for 5 min (min) and then washed out with sterile normal saline before use on wounds.

### Preparation of AgNPs solution

Stable AgNPs less than 100 nm were synthesized by a typical one-step protocol according to Vigneshwaran et al. [43]. One gram (g) of soluble starch was added to 100 ml of deionized water and heated until complete dissolution. One ml of 100 mM aqueous solution of AgNO_3_ crystal (FW. 169, 87 Gamma laboratory chemicals, Assay: Min. 99.0%) was added and stirred well. The mixture was kept into a dark container and autoclaved at 121 °C for 5 min. The formed solution of the AgNPs appeared clear yellow. The stock of the AgNPs solution was kept in dark glass away from direct sunlight and at room temperature (25 °C). The total concentration of the AgNPs stock was measured by the Graphite Furnace Atomic Absorption (210VGP). The size of the AgNPs was measured by the transmission electron microscopy (TEM) (JEOL-JEM- 100CX II).

### Microbiological evaluation of AgNPs-treated fish skin

The AgNPs-treated fish skin strips were swabbed using sterile swabs and bacteriologically counted in four bacteriological media; the Plate Count Agar (HMESIA- Ref -M091A) for enumeration of viable bacteria, Mannitol Salt Agar (Oxoid CM0085) for Staphylococcal account, MacConkey Agar (OMRI W/O Crystal Violet, Biolife) for enumeration of Coliforms, and Difco™ Potato Dextrose Agar (Ref 213,400) for the cultivation of yeasts and molds [44, 45].

### Histological and histochemical evaluation of AgNPs-treated fish skin

Specimens (0.5 × 0.5 cm) were collected from the AgNPs-treated fish skins, as well as, from untreated ones (control). The specimens were fixed in 10% neutral buffered formalin, dehydrated in graded alcohol series, cleared in xylene, embedded in paraffin, and sectioned at 5 μm thicknesses. The obtained sections were stained with Mayer’s hematoxylin (Merck, Darmstadt, Germany) and eosin (Sigma, Missouri, USA) and Gomori’s trichrome stains [46]. The stained sections were examined using a LeitzDialux 20 microscope to evaluate the collagen content according to the depth of the green color, the organization, and the disintegration of the collagen. Using threshold area fraction determination, the percentage of collagen positive area was calculated using the ImageJ software. The amount of collagen was reported as a percentage of the total number of pixels in the optical view as a percentage and expressed as mean ± SD [47].

### Surgical induction of metacarpal wounds

Metacarpal wounds were created under the effect of intravenous anesthesia of 1.1 mg/kg xylazine HCl 2% (Xyla-Ject, ADWIA Co., SAE, Egypt) and 2.2 mg/kg ketamine HCl 5% (Ketamine, Sigma-tec Pharmaceutical Industries, SAE, Egypt) [5].

The metacarpal regions were prepared for aseptic surgery and draped except for the sites of wound creation. Bilateral, circular full-thickness excisional skin wounds (2 cm in diameter) were created on the dorsal aspect of the mid-metacarpals of each donkey using a sterile template. Precautions were taken to avoid injuring of the underlying structures. Hemostasis was achieved by sustained direct pressure over the bleeding sites using a sterile tampon.

Wounds on the right metacarpals (treated wounds, *n* = 9) were dressed with sterile fish skins under sterile non-adherent dressing pads (Siri Pad, Absorbent Dressing Pad, 10 × 20 cm, Elnasr Medical Co., Egypt) and secured by sterile elastic bandages and outer elastic adhesive bandages. Wounds on the left metacarpals (control wounds, *n =* 9) were dressed and secured with the same as above materials and bandages respectively. Donkeys received a prophylactic anti-tetanic serum (3000 IU, subcutaneously) on the day of the surgery.

Wound dressings were changed weekly by fresh prepared AgNPs-treated fish skins and sterile non-adherent dressing pads for treated and control wounds, respectively. Wounds were evaluated microbiologically, grossly, and histologically on days 7, 14, and 21 post-wound inductions in the G7, G14, and G21, respectively. All wounds were allowed to recover following the harvesting of specimens for the histopathological evaluation in donkeys of all groups before releasing them.

### Microbiological evaluation of wounds

Wounds were swabbed with sterile bacteriological swabs on the day of the surgery (days 0), and then on days 7, 14, and 21 post-wound induction for enumeration of microbial contaminants using the four bacteriological media described before. Total bacterial count (TBC), Staphylococcal count, Coliform count (CC), and yeast and mold count were enumerated according to the American Public Health Association (APHA) [44] and Qazi et al. [45].

Reduction percentage was calculated by comparing the microbial counting before and after treatments for each contact time according to the equation of Li et al. [48]: C_0_ – C × 100 / C_0_, where C_0_ is the initial microbial count and C is the count after treatment.

### Planimetry of wounds

Each wound was photographed using a Canon 1XUS 100 IS digital camera, with electrofocus short back focus (EF-S) 5. 9-17.9 mm lens on days 0, 7, 14, and 21 post-wound inductions. A standardized ruler was included in each photograph to allow for digital calibration of the photographs. The distance of the camera was no closer than 30 cm apart from the wound and the wound shape was visible in the middle of the picture. The picture was taken after focusing onto the wound shape. The following measurements were recorded using the ImageJ software analysis as described before [49]:

1. Percentage of wound size at day (x) compared to day (0) = wound size at day (x) mm^2^ / wound size at day (0) mm^2^ × 100
2. Percentage of wound contraction = 100 – the percentage of wound size at day (x)
3. Percentage of epithelialization = size of epithelialization area at day (x) mm^2^ / size of the wound at day (x) mm^2^ × 100

### Histological evaluation of wounds

Wound specimens were harvested under the effect of the anesthetic protocol described before. One 10-mm diameter, full thickness punch biopsy sample was taken from the lateral margin of each wound per time point. The specimens were fixed in 10% neutral buffered formalin and prepared for hematoxylin and eosin staining as described before. The slides were then examined microscopically and the histological evaluation was performed blindly on coded samples. A comparison was made with sections from the untreated wounds. Quantification was carried out to evaluate the epithelial gap (wound length in micrometer from left and right margins), inflammatory cells count, and the average size of the new blood vessels using the ImageJ software. The new blood vessels were counted in 5 images/wound sample [50].

### Histochemical evaluation of wounds

Another set of wound specimens were stained with Gomori’s trichrome stain to evaluate the collagen content as described before [46]. The slides were observed under the microscope and checked for collagen staining with green color. Using threshold area fraction determination, the percentage of collagen positive area was calculated using the ImageJ software. The amount of collagen was reported as a percentage of the total number of pixels in the optical view as a percentage and expressed as mean ± SD [47].

### Statistical analysis

Data were presented as mean ± standard deviation. The statistical analysis was performed using the Graph Pad Prism software version 5.03 (Graph Pad Software Inc., La Jolla, CA, USA). One-way analysis of variance (ANOVA) followed by Tukey’s post hoc test were used to analyze the results of the rate of wound healing. *P-value* < 0.05 was considered statistically significant.

## Data Availability

The datasets used and/or analyzed during the current study are available from the corresponding author on reasonable request.

## Abbreviations

APHA: American Public Health Association
AAZV: American Association of Zoo Veterinarians
AgNPs: Silver nanoparticles
TBC: Total bacterial count
CC: Coliform count
